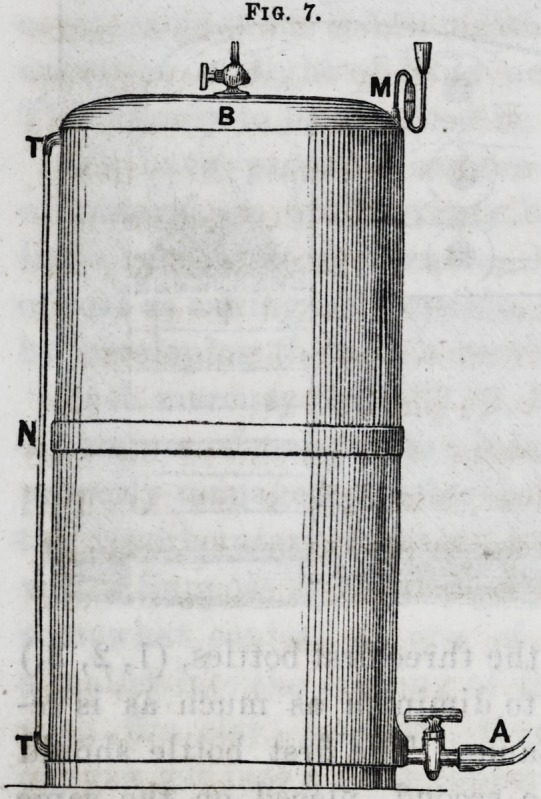# Platina and Its Accompanying Metals

**Published:** 1860-10

**Authors:** H. Sainte Claire Deville, H. Debray


### ARTICLE XII.

*Platina and its Accompanying Metals.* By MM. H. SAINTE CLAIRE DEVILLE and H. DEBRAY. (Translated from the French for the American Journal of Dental Science.)

(Continued from April No.)

### I.—RUTHENIUM.

*Physical Properties.*—After osmium, ruthenium is the most refractory metal of which we have any knowledge. It requires the most vivid jet to melt small quantities of it; and then it is necessary to place the metal at the distance of one or two millimetres from the extremity of the blowpipe, at the point where the heat is at its maximum, or the experiment will not succeed. During this operation the oxyd $RuO_3$ is formed, which volatilizes, emitting an odor resembling that of osmic acid, and depositing a brown sublimate. Ruthenium, after being thus subjected to the oxydating flame is blackish brown upon the surface; it vegetates (*roche*) like platinum and rhodium; it is brittle hard like iridium.

*Density.*—The density of ruthenium is the only property which we find distinctly characteristic of this metal, the color reactions not sufficing for the platinoid metals, one of which, iridium, may present them all. Ruthenium, separated from rhodium by the insolubility of its rose-colored salt, ($Ru_2$ $Cl_3$, 2Clk.,) by its insolubility in the molten mixture of nitre and saltpetre (*sic.*,) is distinguished clearly from iridium only by its density, which is clearly half that of iridium. The purest melted rhodium we have obtained, weighs from 11 to 11.4: it was dissolved a great many times in nitre and potassa, in which iridium becomes insoluble when ruthenium is much in excess, a fact which explains this method of purification.

*Preparation.*—We shall give in succession the widely different methods by means of which we have obtained ruthenium. Osmides in laminæ were selected and pulverized after having been alloyed with four or five times their weight of zinc, which is expelled by heat, employing, as we have already said, a carbon crucible properly protected. For this, the mixed materials are warmed, first to full redness for an hour, then to whiteness for two hours, till all the zinc vapor has disappeared from the flame. In the crucible is found a porous friable mass weighing precisely the same as the osmide originally introduced. Under the pestle, this mass is easily pulverized, except a small quantity of spangles which are separated by a gauze sieve. One part of this finely pulverized matter is mixed, with extreme care, with three parts of binoxyd of barium and one part of nitrate of baryta ; the substance is introduced into an earthen crucible and kept for an hour at a temperature a little below the fusing point of silver. After cooling, the black friable mass is pulverized with the greatest care and introduced into a flask, provided with a ground glass stopper, and filled with a mixture of 20 parts of water and 10 of common muriatic acid. The flask is plunged in cool water to hinder the temperature from rising in consequence of the violent reaction. The newly prepared osmio-iridiate of baryta is added portionwise. This operation must be performed in a good draught, to prevent the small quantities of osmic acid carried off by the disengaged chlorine or oxygen, from diffusing themselves with these gases through the atmosphere of the laboratory. When all reaction is over, one part of nitric acid and then two parts of common concentrated sulphuric acid are added. The flask is then closed, shaken violently, and allowed to stand to deposit the sulphate of baryta. The liquid is decanted, the residue washed by decantation, and the mixed solutions distilled in a tubulated retort, till a fourth of their volume has passed over. The distillate is very rich in osmium, which is immediately precipitated by ammonia and sulphydrate of ammonia. The red liquid remaining in the retort is evaporated to a very small volume. Then two or three parts of sal ammonia are added portionwise and some cubic centimetres of nitric acid. It is evaporated to dryness at a temperature which should not much exceed 100 degrees (212°F.) There remains in the retort a dark violet crystalline precipitate, which is treated with a small quantity of water half saturated with sal ammoniac, and washed with this solution till it is no longer colored. The black salt (chloridiate of ammonia containing ruthenium) is introduced into a porcelain crucible and calcined gradually till the mass is brought to redness. It is well to enclose the porcelain crucible in an earthen one, and to introduce between the two some fragments of charcoal. The mixture of iridium and ruthenium thus obtained, is melted in a silver crucible, with twice its weight of nitre and its own weight of monohydrated potassa, and kept at a dull red for an hour or an hour and a half. It is digested with cold water and the yellowish orange solution of rutheniate of potassa is filtered off by means of a plug of amianthus put in the throat of a funnel. This liquid is treated by carbonic or nitric acid till some bubbles of carbonic acid, of nitric acid or binoxyd of nitrogen are disengaged and all yellow color disappears. It should exhale no odor of osmic acid. It lets fall a precipitate which is oxyd of ruthenium contaminated with a little silica. This oxyd is strongly calcined in a crucible of the coke from gas retorts,* and is melted with great precaution, by means of the oxyhydrogen blow-pipe already described, in a little cup hollowed out of a block

* When the ruthenium contains oxyd of chrome this is transformed in the crucible into brilliant and well crystallized carburet of chrome.

of lime. If the ruthenium contains osmium, chrome or silica, these impurities are disengaged in vapors or in combination with lime.

We give below the details of an operation of this sort:

| | Grammes. |
|---|---|
| Osmide from Colombia in spangles, | 34.10 |
| Mixed with zinc, | 150.00 |
| There remains after volatilization: disaggregated osmium, | 33.95 |
| Portion resisting pulverization, | 0.50 |
| Material employed in the operation, | 30.00 |
| Binoxyd of barium, | 90.00 |
| Nitrate of baryta, | 30.00 |
| Muriatic acid for solution, | 300.00 |
| Material escaping solution, | 0.00 |
| Murohydrated sulphuric acid for precipitation of the baryta, | 60.00 |
| Iridium and ruthenium oxydated, | 20.50 |
| Reduced by hydrogen, | 19.25 |
| Potassa, | 20.00 |
| Nitrate of potassa, | 40.00 |
| Ruthenium reduced and freed from silica, | 1.85 |

This ruthenium should be purified by one or more fusions with nitre and potassa, till the density is about 11.3

*Process by Roasting.*—It is known that M. Fremy has given a very elegant process for obtaining crystallized oxyd of ruthenium. But this process succeeds only with difficulty in a very numerous class of ores, which containing only a small proportion of ruthenium, furnish it in small quantities. It is manifest that in operating upon ores rich in ruthenium, this is the mode of preparation which should first be employed, in order to extract in a crystallized form, all that can be furnished by this process, the residue being afterwards treated by the methods we have just indicated. The processes by roasting, applied to ruthenium, are so simple, that to obtain specimens, we always employ them, when the osmides roast readily. When on the contrary, calcination is difficult, it is well, as we have said when speaking of the preparation of osmic acid, to treat the osmide of iridium with 7 or 8 times its weight of zinc, to dissolve out with muriatic acid all the excess of zinc, to calcine the powder to dull red in a closed crucible, and finally to effect the calcination at the temperature of the fusion of copper, on platina foil in a tube of porcelain. We have thus obtained fine crystals of oxyd of ruthenium in prisms with a square base of the form of oxyd of tin.

*Oxyd of Ruthenium*, $RuO^2$.—Thus obtained, this oxyd has a density of 7.2 and the following composition:

| | |
|---|---|
| Osmium oxydated, | 0.7 |
| Iridium oxydated, | 1.0 |
| Rhodium oxydated, | 1.0 |
| Oxyd of ruthenium, | 97.3 |
| | 100.0 |

This analysis was made by dissolving the oxyd in potassa and nitrate of potassa, and receiving upon the residues the same treatment till all coloration ceases.

This oxyd contains

| | Observed. | | Calculated. |
|---|---|---|---|
| Oxygen, | 22.3 | $O_2$ | 23.3 |
| Ruthenium, | 77.7 | Ru | 76.7 |

These are the results also obtained by M. Fremy. The immediate analysis of this oxyd shows plainly the cause of differences between the figures calculated for oxygen and those given by observation.

We call attention to the fact that all the colored reactions of ruthenium belong almost without exception to iridium, that the composition of the oxyd of ruthenium is such, that it might as well represent an oxyd of iridium, $IrO_4$ as the oxyd $RuO_2$, and that because the equivalent of ruthenium is plainly half of the equivalent of iridium ; we have shown that the oxyd of iridium dissolves easily in the mixture of nitre and of saltpetre (*sic*:) it was, therefore, indispensable to show that this volatilized oxyd was not a new degree of oxydation of iridium. We have accomplished this in the following experiments:

With this oxyd we have formed the rose-colored salt of M. Claus, which has given us by analysis, the following numbers:

| | Observed. | | Calculated. |
|---|---|---|---|
| Chloride of potassium, | 40.0 | 2ClK | 40.9 |
| Chlorine, | 30.2 | 2Cl | 29.6 |
| Ruthenium, | 29.7 | 2Ru | 29.5 |
| | 99.9 | | 100.0 |

The metal roasted in the air becomes the protoxyd, which has the following composition:

| | Observed. | | Calculated. |
|---|---|---|---|
| Ruthenium, | 85.9 | Ru | 86.8 |
| Oxygen, | 14.1 | O | 13.2 |
| | 100.0 | | 100.0 |

Finally, after all these experiments, the metal which was disengaged from this last compound, had a density very near 11.3*, which characterises it in the most precise manner, for iridium weighs, 21.15.

Furthermore, we call attention to the fact that the density of the molten matter is the best criterion of the purity of ruthenium. The mixture of potassa and saltpetre dissolves so many substances different from ruthenium, that to be sure we have impure ruthenium, we are obliged to have recourse to this method of verification. It is thus that ruthenium not purified gives variable densities, ranging from 17 to 14† and is never free from iridium. Fur-

*This density, taken upon a very small quantity of material, can only serve as a verification.

†The ruthenium of density 14 contains

| | |
|---|---|
| Ruthenium, | 88.7 |
| Iridium, | 11.3 |
| | 100.0 |

ther, if we take, as we have done, osmides in grains entirely free from ruthenium, and attack them by the mixture of nitre and potassa, we obtain a deep blue liquid from which is easily extracted a metal, which, melted and refined, has a density of 21.15, like pure iridium. Thus in attacking 32.3 grammes of metallic iridium from osmium, in grains, we have obtained 4.43 grammes, or 14 per cent. of a metal soluble in the alkaline flux, which after fusion weighed 21.2. It possesses besides all the colored reactions of iridium.

*Alloys of Ruthenium.* Zinc forms with ruthenium an alloy which presents itself under the form of hexagonal prisms, probably regular, formed at the end of an almost complete evaporation of the zinc. This alloy takes fire in the air and burns with feeble deflagration. Its composition could not be determined for want of material.

The alloy of ruthenium and tin crystallizes in cubes, the angle of which has been found to be exactly 90 degrees. It is perhaps the finest alloy which can be produced ; comparable to the finest specimens of crystallized bismuth, in the beauty and size of its crystals. It is very easily prepared. It is sufficient to heat to redness in a charcoal crucible, ruthenium with ten or fifteen times its weight of tin, and to attack the cooled matter by muriatic acid. We find a geode of superb crystals, the composition of which is

| | | | |
|---|---|---|---|
| Ruthenium | 33 | Ru | 31* |
| Tin | 67 | 2Sn | 69 |
| | 100 | | 100 |

We cannot conclude these remarks without rendering homage to the sagacity and precision with which the discoverer of ruthenium, M. Claus, has treated this subject, on which he would have left nothing to be done, if he had

* The ruthenium employed was not pure enough to hope for more closely corresponding numbers.

had at his disposal the dry methods which we have employed almost exclusively. Still we know by experience how difficult this subject is, especially when it is wished to obtain results as precise as those which are to be found in the beautiful memoirs of M. Claus.

### II.—Palladium.

Palladium is the most fusible of all the platinoid metals. The furnaces which serve for the fusion of platinum bring it to the liquid state with extreme facility. When we subject it, by means of an explosive gas blowpipe, to the temperature of fusion of iridium, it disappears in green vapors which condense into a dust of a bistre color, a mixture of the metal and its oxyd.* This experiment should be made in a little cupel sunk in a piece of quick lime.

Palladium, heated in contact with the air and kept in fusion in an oxydating atmosphere, vegetates like silver at the moment of its solidification. The oxygen disengaging itself only at the moment when the upper layer of the metal is fixed, the button which has vegetated is cavernous although its surface is perfectly regular. Palladium, closely allied to silver, is more oxydizable at a low temperature. Its surface is always tarnished by a very slight layer of oxyd.

When we wish to make with palladium the experiment of the flameless lamp, we must begin by heating its surface in the reducing flame. These experiments succeed very well, if a plate of palladium is put in a current of illuminating gas mixed with air, as it comes from the me-

* When we subject silver to so elevated a temperature, taking care to maintain the oxygen in slight excess in the flame, we see the silver boil like mercury and disappear in fumes of oxyd, which can be condensed on the fragments of the crucible or on the lime in which the little cup has been scooped out for the experiment. The oxyd of silver thus produced is clear yellow, like a sublimate of lead, but not so deep in tint.

tallic gauze of a common lamp. With this lamp the metallic lamina is warmed, then the flame is extinguished by turning off the gas. Some moments after, the metal being still a little hot, the gas is let on and the palladium becomes incandescent.

The density of pure palladium, melted and not hammered, is 11.4 at the temperature of 22.5° (75.5 F.)

*Alloys of Palladium.* Palladium is soluble in zinc, but does not combine with it: for, after the action of muriatic acid on an alloy of zinc and palladium, only palladium is found. With tin it is otherwise. On melting palladium with six times its weight of tin, heating to redness, cooling and digesting with muriatic acid, there remains a compound crystallized in fine brilliant laminæ, which has the following composition:

| | | | |
|---|---|---|---|
| Palladium | 57.4 | $Pd_3$ | 57.4 |
| Tin | 42.6 | $Sn_3$ | 42.6 |
| | 100.0 | | 100.0 |

Silver and copper, which have a great analogy with palladium, give by this process alloys with tin altogether similar in form and composition.

| | | | Calculated. | | | | Calculated |
|---|---|---|---|---|---|---|---|
| Silver | 73.7 | $Ag_3$ | 73.3 | Copper | 44.8 | $Cu_3$ | 44.9 |
| Tin | 26.3 | $Sn_2$ | 26.7 | Tin | 55.2 | $Sn_2$ | 55.1 |
| | 100.0 | | 100.0 | | 100.0 | | 100.0 |

### IV.—RHODIUM.

It is easy to get rhodium by attacking the residues of platina by any process and especially by that of M. Woehler, that is to say, by means of chlorine which has been made to act on a mixture of common salt and the residues, precipitatiug the iridium by sal ammoniac, and searching for the rhodium in the soluble products.

Rhodium reduced by hydrogen should be purified in the manner we shall presently describe. The following is the complete process we recommend.

There is among platina residues a particular substance in which it is best to look for rhodium. It is that which is obtained in workshops when the mother waters, from which platina has been thrown down, are precipitated by iron. We shall presently give, in the chapter on platina residues, the composition of these substances. We shall content ourselves, at present, with describing the general process by which we extract pure rhodium from every variety of residue. We begin first by melting these residues with their weight of lead, and twice their weight of litharge. When the crucible in which the operation is performed is thoroughly red, the litharge completely liquid, it is agitated once or twice and suffered to cool slowly. The wedge of lead is then withdrawn and thoroughly cleaned ; it contains all the less oxydizable metals belonging to these residues. The lead is attacked by nitric acid diluted with its weight of water, which dissolves the lead, the copper and the palladium. The pulverized metallic substance which remains is well washed, then mixed with extreme care with five times its weight of pulverized binoxyd of barium which is exactly weighed. The mixture introduced into an earthen crucible, is kept at redness for one or two hours, taken up by water, then by aqua regia which expels a great quantity of osmium that is lost, or may be recovered by distillation in the state of osmic acid. When the liquid has lost all odor, a quantity of sulphuric acid is added, sufficient wholly to expel the baryta from the mixture of the chlorides. It is boiled, filtered, evaporated, with the addition at first of a little nitric acid, and then, at the end of some time, of a great excess of sal ammoniac. It is evaporated to dryness by warming it to 100° (212° F) and washing with a concentrated solution of sal ammoniac, which carries off all the rhodium, the washing being continued till the wash waters are no longer sensibly tinted with rose. The filtered liquor is evaporated with a great excess of nitric acid which destroys the sal ammoniac, and, when there remains only the salt of rhodium, the evaporation is finished in a porcelain crucible. The dry matter is moistened with a little sulphydrate of ammonia, and mixed with three or four times its weight of sulphur. The porcelain crucible with its cover on is then introduced into an earthen crucible and the interval between the two is brasqued. The whole is heated to bright redness and there remains in the crucible metallic rhodium, which may be considered as very nearly pure when it has been for a long time boiled successively with aqua regia and concentrated sulphuric acid.

To obtain rhodium with the properties we are about to describe, it must undergo another purification. Indeed, this rhodium forced up by the hammer, exhibits well the equivocal malleability attributed to it; but once melted, it will lose it almost entirely in consequence of the incorporation into the entire mass of the impurities mechanically mixed with the metal. To obtain irreproachable rhodium, it is mixed with three or four times its weight of zinc, melted at dull redness, thoroughly stirred, then left awhile to rest and poured out. At the moment when the alloy is made, there is developed such a heat that a part of the zinc may be volatilized; the crucible must then be covered with the greatest care. The alloy treated with concentrated muriatic acid, gives up much of its zinc, leaving a crystalline substance which is nothing but an alloy of definite proportions of zinc and rhodium. This is dissolved in aqua regia; the solution treated with an excess of ammonia to complete or nearly complete solution of the precipitate.* After ebullition for some time and a proper

* This mode of preparation by ammonia and crystallization of the chloramidide of rhodium is applicable to all solutions of rhodium containing little iridium. The zinc which we add to the rhodium to facilitate its solution is a evaporation, the yellow salt or chloramidide of rhodium* is obtained. This, after two or three crystallizations, and calcination with a little sulphur in a charcoal crucible, at a high temperature, gives pure, agglomerated rhodium which may be then melted without loss.

The fusion of rhodium can be effected either by means of the blow-pipe we have described and a little cupel in lime, or in the lime which answer for the fusion of platina and which will presently be described.

Rhodium melts less readily than platina, so that the same fire which liquifies 300 grammes of platina, can fuse in the same time only 40 or 50 grammes of rhodium. We have observed no appearance of volatility in this metal; but it oxydates very superficially like palladium and vegetates in the same manner. The surface of the button is often bluish. When the rhodium has been thus melted in contact with lime, it is deprived of the silicium which always accompanies it and of the osmium of which the last traces disappear only at a very high heat, and it acquires physical properties eminently utilizable. Less white and less brilliant than silver, it has nearly the same tint as alluminium. It is ductile and malleable, but only in a condition of great purity, at least after fusion. This is a test which the rhodium we find in commerce does not always stand, and which nevertheless in the state of condensed sponge seems capable of being worked with some facility.

The density of pure molten rhodium is about 12.1.

volatile reagent which will not resist the action of fire, when the rhodium is melted; so that we need not trouble ourselves about its possible presence in the yellow salt which has been crystallized two or three times.

* The analysis of this salt gives us the following results which confirm the analysis of M. Claus.

| | Found. | | Calculated. |
|---|---|---|---|
| Rhodium | | 2 Rh | 35.2 |
| Chlorine } | 34.6 | 3 Cl | 36.0 |
| Ammonia } | 65.4 | 5 $NH_3$ | 28.8 |
| | 100.0 | | 100.0 |

*Alloys.*—The alloys of rhodium, at least those which we have examined, are very curious in the fact that they are true chemical compounds, as the high temperature developed at the moment of their formation might have led us to suspect.

We have already described the preparation of the crystalline alloy of zinc and rhodium. It resists the action of muriatic acid; but it is a remarkable fact that upon the contact of air with the acid, there is soon a well marked rose tint, revealing an oxydation of the two metals under the double influence of the air and the acid.

Its composition is

| | | | |
|---|---|---|---|
| Rhodium, | 43.7 | Rh. | 44.5 |
| Zinc, | 56.3 | $Zn_2$ | 55.5 |
| | 100.0 | | 100.0 |

In the same manner is prepared an alloy of rhodium and tin. Muriatic acid leaves a brilliant black crystallized substance, fusible at a high temperature, and easily analyzed by means of the action of chlorine, which transforms it into volatile chloride of tin, capable of being weighed as stannic acid and rose-red chloride of rhodium, which is fixed, insoluble in aqua regia, and having the composition RhCl.: this can be weighed as metal by reducing it with hydrogen. We give the following results:

| | | | Calculated. |
|---|---|---|---|
| Tin, | 53.2 | Sn. | 53.1 |
| Rhodium, p. d. | 46.8 | Rh. | 46.9 |
| | 100.0 | | 100.0 |

### V.—Platina.

After palladium, platina is the most fusible metal of the group. Once melted, if the temperature is much raised and its action on the button prolonged, the metal sensibly volatilizes. It presents, at the moment of its solidification, the phenomena of vegetation, which heretofore has been observed only for silver. To make platina vegetate, the operator must keep in fusion in lime for a long time a mass of at least 500 or 600 grammes, and rapidly uncover the metallic bath. When allowed to cool slowly, platina does not vegetate.

The best method of procuring pure platina is to melt and *refine* it in lime. We find in commerce platina of the second and third solution which is almost free from iridium, but which always contains traces of osmium and a little silicium. Fusion in lime in the oxydating flame perfectly refines it; osmic acid is liberated and the silicium passes into the state of silicate of lime, which melts to a colorless pearl that is seen to move rapidly over the surface of the metal till it reaches the edge, and is absorbed by the walls of the furnace.

Melted platina is a metal as soft as copper, as has been fully proved at the Paris mint: it is whiter than common platina, and does not possess that porosity which has hitherto hindered the fabrication of an impermeable vessel of platina.

Melted platina still possesses the property of condensing gases on its surface, and producing the phenomena of the flameless lamp.

Its density is 21.15; less than that of ordinary platina which has undergone, in working, very powerful crushing.

*Fusion of Platina.*—We are about to describe that apparatus with which we have succeeded in melting platina, while operating upon quantities relatively large, and casting it in an ingot-mould, like metal of ordinary fusibility.

The combustible we used most frequently is illuminating gas. Hydrogen, which, at least when pure, gives a still greater heat, may also be employed. The combustion is fed by a current of oxygen, and the distribution of the gases is accomplished by the blow-pipe, (fig. 2,) which we shall not again describe. We shall only remark that to melt sufficient quantities of platina, 12 or 15 kilogrammes for example, that the stop-cocks of the apparatus, especially that which carries the coal-gas, should have a considerable section, leaving a square centimetre, or at least 75 square millimetres for the passage of the gas. The platina *tip* of the blow-pipe, or rather the hole by which the oxygen escapes, should be at least two millimetres in diameter. The oxygen ought to be under a pressure of from four to ten millimetres of mercury.

The furnace (fig. 3,) in which the combustion is made, is lime encircled with threads of iron. It is composed of two parts: 1st, the dome A A, cut in a cylindrical piece of lime, slightly arched at its lower part and pierced with a conical hole, where the blow-pipe C E enters: second, of a sole B, hollowed in another cylindrical piece of lime. This should be so deep that the molten platina may occupy a thickness of at most three or four millimetres. At the anterior part D, which should slightly protrude, a groove slightly inclined inward, is made with a file. This ought at the same time to serve as a spout for pouring, and an outlet for the flame. To effect a fusion, the different pieces of lime constituting this apparatus, are arranged in the manner represented in the figure; then holding the blow-pipe in the hand, the stop-cock H, (fig. 2,) is opened, and a feeble current of combustible gas allowed to pass, and then, by turning the stop-cock O, sufficient oxygen is let on to burn it. The flame is introduced through the hole P, so as to avoid a slight explosion which might scatter the lime of the apparatus. The walls of the furnace are gradually heated by slowly augmenting the rapidity of discharge of the gas, till the maximum of temperature has been obtained. With a piece of platina foil introduced at D, (fig. 3,) and placed under the jet of gas, we can ascertain the point of maximum temperature,

Fig. 3.

that is, the point at which fusion goes on with greatest rapidity; this is lowered or raised as required by managing the screw P, (fig. 2,) and lowering or raising the orifice of the platina tip which lets in the oxygen. The screw is kept steady and platina gradually introduced at the aperture D. If the platina is in small scales of less than a millimetre in thickness, there is scarcely time to introduce them. They are seen to disappear and melt almost the moment they enter the furnace. The oxygen should have a certain pressure, (about four or five centimetres of mercury,) and should agitate the platina with a gyratory motion, which regulates the temperature throughout the mass.

When it is not intended to cast the platina, the fusion being complete and the refining finished, which is known by the formation of vitreous matter on the surface ceasing, the rapidity of the current of the two gases is diminished, allowing the reducing gas to be very slightly in excess. This gas determines a very rapid formation of water or carbonic acid, at the expense of the combustible gas and the oxygen dissolved in the platina, and then a very perceptible ebulition is observed in the metallic mass. Gradually the solidification is effected even to the centre, and the fire is entirely extinguished. There is always a projection of platina towards the roof of the furnace, but this is easily collected after the operation.

When the platina is to be cast, an ingot mould is prepared either of thick cast iron well rubbed with plumbago, or of coke of gas retorts, or of lime. The last are made very readily of separate pieces of the material bound with iron wire. The vault is removed, the bed seized with the pincers, and the platina leisurely poured out like ordinary metal. The only difficulty (and that practice enables us to surmount) is to distinguish the dazzling surface of the platina and the open mouth of the mould, so as to pour steadily. The attempt should never be made to cast more than thr ee or four kilogrammes at once. The danger is too great, should the pincers or any other part of the apparatus give way. It is necessary (as we have done when operating upon 12 kilogrammes of platina at a time) to use a furnace (see fig. 4) constructed according to the principles

Fig. 4.

we have just described, but composed, on account of its size, of pieces of lime arranged like bricks in a cylindrical apparatus of iron plate, in which they are readily disposed, the sole K being afterwards scooped out. The vault V is composed of several pieces of lime bound by a very solid circle of iron, supplied with a pressure screw ; when the pieces are well set and tightened, the surface of the vault is dressed and the hole Q pierced with the greatest facility. The sole contained in the cylinder of iron plate K turns on a hinge about two supports LL, so arranged that the horizontal line joining the hinges, passes through the taphole D, so that when it is lifted by the handle S, attached to the iron cylinder, the whole apparatus turns about the line

LL, and the liquid on the sole of the furnace, runs into D, without displacing the point D itself. This very simple little manœuvre can be practiced by filling the sole with mercury, and pouring out this metal before trying melted platina. The principles upon which we base our apparatus are very simple.

1. Lime is perhaps the worst known conductor of heat, so that through a thickness of, at most, 2 centimetres, the apparatus being full of molten platina, the exterior is hardly 150 degrees, (302° F.)

2. Lime is the body which radiates heat and light with greatest perfection, whence it has been selected for the Drummond light. These are, therefore, the best walls that can be selected for a reverberating furnace of this kind.

3. Lime acts on all impurities which we need to separate from platina, such as iron, copper, silicium, &c., and transforms them into fusible combinations which penetrate its porous substance. It acts like a cupel, the substance of which purifies the metal melted in it.

An experiment made in the laboratory of the normal school with gasometers of 1400 to 1500 litres of oxygen and illuminating gas, by means of the apparatus with hinges, gave the following results :

In forty-two minutes, in which is comprised the time necessary for the determination of the proportions of gases necessary, and the trials inseparable from the manipulation of an apparatus which was not yet thoroughly understood, we have melted 11.595 kilogrammes of platina Russian coin. After the fusion it was necessary to refine the metal, which contained a little osmium and notable quantities of silicium, and then to run it in an ingot-mould of gas-coke, where it was kept liquid a considerable time. The amount of oxygen used was 1200 litres and the loss of weight in platina 135 grammes, half of which consisted of mechanical losses by projection during the pouring, so that the real loss is estimated at ½ per cent. at most, of the weight of platina; which corresponds very nearly to the impurities contained in the metal.

Thus, including refining, each kilogramme of platina requires for its fusion 100 litres of oxygen gas. But the refining requires almost as much as fusion; for in experiments of this kind made with very pure platina belonging to M. Savard, operating on three kilogrammes, a quantity already too small for such a determination, the amount of oxygen necessary to melt each kilogramme of platina, was only sixty litres.

The casting of platina requires the same precautions as that of silver. According to the trials made with the tools of M. Savard and his platina, which we have often melted, (thanks to his very obliging disposition,) we have had with the same material badly formed ingots, malleable, but blebby, and often an unobjectionable substance, comparable to the softest metal. There is, therefore, in this operation a slight of hand necessary for certain success. This is only to be obtained by practice.

We have prepared oxygen by means of binoxyd of of manganese and mercury bottles which we heated in a little reverberatory furnace, (fig. 5,) with a thick layer of coal as caking as possible, to avoid burning the bottles by enveloping them in a very smoky flame.

Each mercury bottle (1, 2, 3, 4, 5, 6,) contains five kilogrammes of manganese; it lasts almost indefinitely when properly managed. After being charged, it is placed in the reverberatory furnace, horizontally or vertically, at will. Suppose it is charged horizontally—an iron tube somewhat conical at one of its extremities, driven by a mallet into the opening of the mercury bottle, and luted with fire clay, conducts, through caoutchouc tubes, the oxygen gas into a little copper receiver (fig. 5) containing water, and receiving the tubes coming from the mercury bottles, so as to isolate them from one another by means of the layer of water which the gases are forced to traverse. On the lateral and upper part of the receiver, a tube first horizontal and then slightly inclined, carries the gases and condensed water into a flask full of slacked lime or of a solution of caustic soda, in which the oxygen loses its carbonic acid. This vessel ought to be cooled if the receiver is not, because the water which the peroxyd of manganese, arriving there as steam, might heat it excessively. Thence the gas passes into the gasometer full of water, of which the discharge is so regulated that the pressure ought always to be some centimetres of water higher than that of the atmosphere.

The form of the reverberatory furnace, which we employ at the normal school is so simple that there is no need to describe it. Fig. 5 will answer in place of a description.

FIG. 5.

We shall only observe that the three first bottles, (1, 2, 3,) ought to be close together to diminish as much as is required the rapidity of the flame. The first bottle should nearly touch the vault; the second, placed on the same vertical line, ought to be very near the first, and the third should only leave between it and the sole of the furnace the space necessary to give passage to nearly all the flame. After this the three others are regularly disposed in the remaining space. It must always be remembered that the flame and heat tend always to rise, and that our object should always be to keep them down.

Fig. 6.

Fig. 6 indicates the disposition of the receiver with its tubes, AA, for the admission of the gas; the hole of exit, B, which determines the level of the water, and the manometer M, which gives the pressure of the gas. It is cooled by a current of cold water.

Fig. 7 gives the construction of our gasometers which we make of zinc in a very simple form. The exit pipe for the water, A, serves also for the introduction of the water to drive out the gas, when it is to be used. It should have a great section; and for a gasometer of 800 litres it should be at least two square centimetres in section. The stop-cock, B, serves also alternately for the introduction and the exit of oxygen gas The manometer, M, contains mercury, and indicates the pressure while the gasometer empties itself. Finally a glass tube, N, connected by caoutchouc tubing with two little lateral tubulures, TT, communicating with the interior of the gasometer, allows us to determine the level of the water.

Fig. 7.

The price of the oxygen is very easily calculated : 100 kilogrammes of German manganese of 75 per cent. costs 26 francs, and it is best to take this highly esteemed article, because after having served for the manufacture of oxygen, the manganese being as good if not better than before for the glass makers, can be sold for 10 francs the 100 kilogrammes, which makes little more than 17 francs per 100 kilogrammes of manganese. According to our experiments, 25 kilogrammes of manganese at 26 francs, give 1500 litres of oxygen, or 100 kilogrammes of manganese gives 6 cubic metres or 8.6 kilogrammes of oxygen.

It is seen that in practice we have obtained about the third of the oxygen which 100 kilogrammes of manganese at 75 per cent. (or 75 kilogrammes of pure manganese) contain, that is, very nearly the theoretic quantity. We have used very rich specimens of manganese, and dearer in proportion than the poor manganese, which it might be better to employ, because the heating and the vessels have a very low value, relatively to the price of manganese. According to these experiments, every cubic metre costs three francs, reckoning the manganese at 17 francs the 100 kilogrammes, and rating at a high price the fuel, the vessels, &c., we get four francs the cubic metre. The cost of fusing a kilogramme of refined platina will therefore be at most 40 centimes, and a kilogramme of pure platina, 24.

The quantity of illuminating gas employed to melt 11.590 kilogrammes of platina, was only a few hundred litres, so that it need not be taken into account, especially when the figures cannot be more exact than is common with laboratory experiments on a small scale.

If hydrogen is used, we have said we get a higher temperature. We prepare the hydrogen in an apparatus of Desbassayns of Richmond, containing 60 litres, in which are placed 100 kilogrammes of zinc, by means of a flask, or balloon of glass or of earthenware tubulated below, containing 50 or 60 litres, and exactly filled with scraps of zinc. A tube carries sulphuric acid to the top of the zinc, and the sulphate of zinc runs out through a tube of copper provided with a stop-cock, also of copper, which must be washed after every operation. The opening of this stop-cock is so regulated that the saturated solution of zinc, after having traversed the layer of metal in the balloon, cannot accumulate in it. A glass tube, communicating above and below with the interior of the balloon, gives the level of the liquid which it contains. The balloon should be constantly sprinkled with cold water, to hinder a rise of temperature. This point is very important. To the cork in the upper part of the balloon is adapted a curved tube, plunging into an eprouvette full of water, and 20 or 30 centimetres high. It is a safety valve through which the hydrogen escapes, when its discharge is suspended or even retarded. The hydrogen generator may be lead, or copper sheathed in lead. It is well, then, to plunge it entirely in a great trough of water, which is constantly renewed. It is necessary, at all hazards, to prevent the water condensed in the tubes which bring the gas to the blow-pipes, from remaining there, as it would occasion oscillations in the pressure, the effect of which would be very injurious to the operation. We repeat it again—all the stop-cocks, all the channels for conducting the different gases, should be of a large section.

The method which we have just expounded, applied to the revivification of old platina, gives excellent results. No foreign metals, except iridium and rhodium, can exist in the platina after it has been melted and refined by the processes which we have described. All the substances which most easily attack platina, sulphur, phosphorus, arsenic, the gold with which it is soldered, iron, copper, palladium, osmium, are separated either by oxydation and absorption by the lime, or by volatilization.

Platina containing gold and palladium, allows these metals to escape as vapor, and they can be easily collected again by carrying the products of combustion through an earthen channel, where all the volatile foreign matters are deposited, except the osmic acid, which is condensed, if a vessel full of ammonia is placed in the current of the vapors. Besides, a part of the osmium is deposited in the tube in the metallic form, being either volatilized in the current of flame, or reduced in the tube of condensation from the osmic acid produced in the furnace.

The form of the furnaces which we have employed might be slightly modified, for example, made elliptical or rectangular, if the fusion were to be accomplished by two blowpipes. It is evident that the quantities of platina which can be melted are unlimited; which has struck all persons who have assisted at our experiments, and who have proved with us that platina is very easy to melt, to run, and even to cast; for platina remains a long time melted in a lime mould previously heated to 500 or 600 degrees. Besides, platina can be melted in separate furnaces, containing 25 to 50 kilogrammes, and poured out at the same time into the same mould, as is done with steel. We only recommend never to give more than 4 or 5 centimetres of thickness to the bath of platina, or at least, that it should be continually agitated either by the blast of the blowpipes, or by stirrers of well burned lime or magnesia, because platina is not a sufficiently good conductor to remain perfectly liquid under a greater thickness. Otherwise, we might fail in refining or even in fusing the metallic mass.

*Alloys of Platina.*—Tin and platina form an alloy well crystallized in cubes, at least in rhombohedra, the angles of which are very nearly 90 (the faces have always little lustre;) their composition is expressed by the formula.

| | Observed. | | Calculated. |
|---|---|---|---|
| Platina, | 52.9 | $Pt^2$ | 52.6 |
| Tin, | 47.1 | $Sn_3$ | 47.4 |
| | 100.0 | | 100.0 |

It is obtained by melting platina with six times its weight of tin, allowing it to cool slowly, and dissolving in muriatic acid the excess of tin, which leaves the alloy of platina in very beautiful and elegant geodes.

The alloys of platina with the common metals * have almost all been studied, and offer but little interest. Zinc appears to combine with platina, and form a crystallizable alloy, which is obtained by melting platina with an excess of zinc, and attacking by muriatic acid, which dissolves the excess of zinc. It is very difficult to hinder a little platina from escaping the action of the zinc, whence, in our analysis, the platina has always been found in excess over the numbers which the formula $Pt_2$, $Zn_3$ gives, showing its composition to be,

| | |
|---|---|
| Zinc, | 30.4 |
| Platina, | 69.6 |
| | 100.0 |

* The metals of platina being infusible by methods hitherto adopted, it has been impossible to make serious attempts to obtain true alloys with them. Still, M. Chapuis has shown us an alloy of platina and rhodium from the aggregation of sponge, and appearing homogeneous ; we have melted it much more easily than rhodium. After refining, which carries off silicium and a little osmium, the alloy is easily worked. According to the observations of M. Chapuis, it is not attacked by *aqua regia*, which makes it one of the most precious materials for the manufacture of certain chemical vessels. This alloy ought to contain 30 per cent. of rhodium. The other alloys will be described hereafter.

[To be Continued.]

## Figures and Tables

**Fig. 3. f1:**
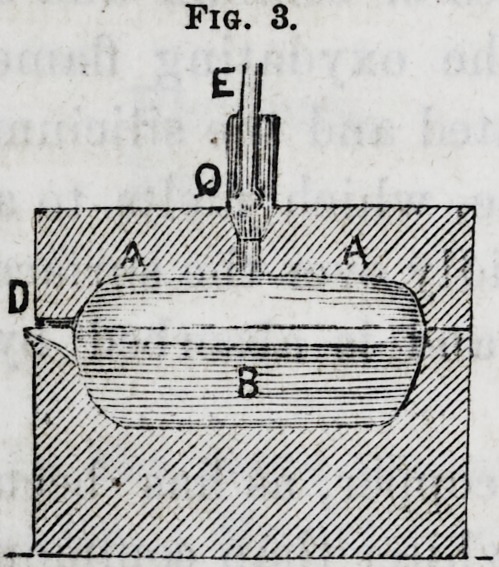


**Fig. 4. f2:**
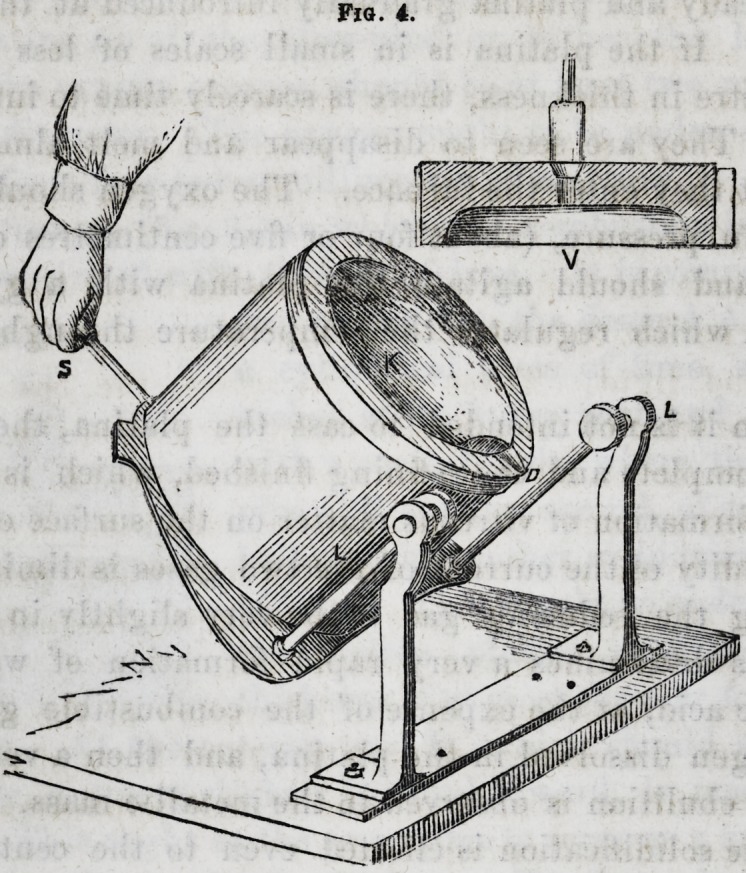


**Fig. 5. f3:**
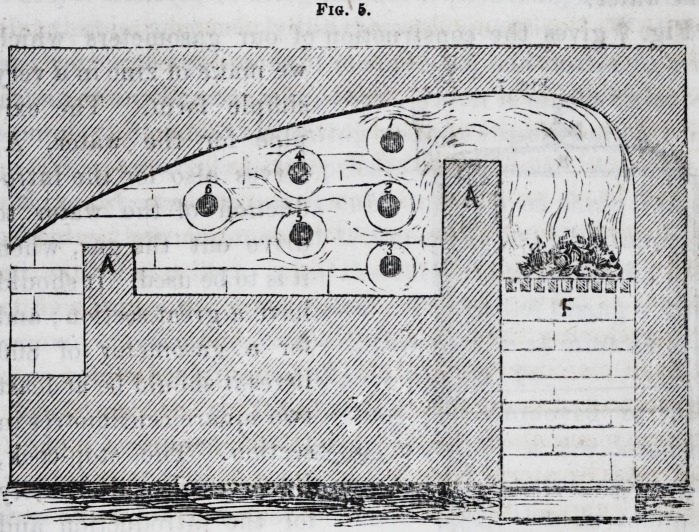


**Fig. 6. f4:**
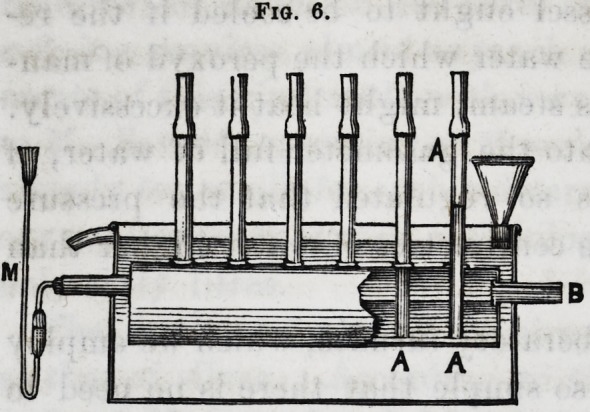


**Fig. 7. f5:**